# Relationship Between Autophagy and Metabolic Syndrome Characteristics in the Pathogenesis of Atherosclerosis

**DOI:** 10.3389/fcell.2021.641852

**Published:** 2021-04-15

**Authors:** Jing Xu, Munehiro Kitada, Yoshio Ogura, Daisuke Koya

**Affiliations:** ^1^Department of Diabetology and Endocrinology, Kanazawa Medical University, Uchinada, Japan; ^2^Department of Endocrinology and Metabolism, The Affiliated Hospital of Guizhou Medical University, Guiyang, China; ^3^Division of Anticipatory Molecular Food Science and Technology, Medical Research Institute, Kanazawa Medical University, Uchinada, Japan

**Keywords:** autophagy, inflammation, oxidative stress, atherosclerosis, metabolic syndrome, type 2 diabetes

## Abstract

Atherosclerosis is the main cause of mortality in metabolic-related diseases, including cardiovascular disease and type 2 diabetes (T2DM). Atherosclerosis is characterized by lipid accumulation and increased inflammatory cytokines in the vascular wall, endothelial cell and vascular smooth muscle cell dysfunction and foam cell formation initiated by monocytes/macrophages. The characteristics of metabolic syndrome (MetS), including obesity, glucose intolerance, dyslipidemia and hypertension, may activate multiple mechanisms, such as insulin resistance, oxidative stress and inflammatory pathways, thereby contributing to increased risks of developing atherosclerosis and T2DM. Autophagy is a lysosomal degradation process that plays an important role in maintaining cellular metabolic homeostasis. Increasing evidence indicates that impaired autophagy induced by MetS is related to oxidative stress, inflammation, and foam cell formation, further promoting atherosclerosis. Basal and mild adaptive autophagy protect against the progression of atherosclerotic plaques, while excessive autophagy activation leads to cell death, plaque instability or even plaque rupture. Therefore, autophagic homeostasis is essential for the development and outcome of atherosclerosis. Here, we discuss the potential role of autophagy and metabolic syndrome in the pathophysiologic mechanisms of atherosclerosis and potential therapeutic drugs that target these molecular mechanisms.

## Introduction

Atherosclerosis is the main cause of mortality and morbidity in metabolic-related diseases, including cardiovascular disease (CVD) and type 2 diabetes mellitus (T2DM) (Weber and Noels, 2011; Barquera et al., 2015; Schneider et al., 2016). The formation of atherosclerotic plaques is divided into four stages: fatty streaks, atheromatous plaques, complicated atheromatous plaques and clinical complications (Hassanpour et al., 2019). Rupture of plaques may lead to an acute occlusion of artery, myocardial infarction or stroke. Three types of cells, endothelial cells (ECs), vascular smooth muscle cells (VSMCs) and monocytes/macrophages, participate in the development of plaques. Lipids and multiple inflammatory cytokines accumulate in the vascular wall. Monocytes migrate to the endothelium of blood vessels, enter into the inner membrane then proliferate and differentiate into macrophages. In this process, monocytes combine with lipoproteins to form foam cells. In smooth muscle cells (SMCs), with the secretion of fibrous elements, the accumulation of fatty streaks and the production of extracellular matrix, plaques develop and increase in size gradually. When macrophages and SMCs die, the necrotic core of the lesion rich in lipid will be formed. Meanwhile, matrix metalloproteinases and neovascularization secreted by macrophages weaken the stability of fibrous plaques. Once plaque rupture, the recruitment of platelets will be initiated to form thrombus (Lusis, 2000; Glass and Witztum, 2001; Hassanpour et al., 2019).

Metabolic syndrome (MetS) is defined as a series of chronic metabolic disorders. Although the details and criteria of the definition differ among different associations, such as the World Health Organization (WHO) (Alberti and Zimmet, 1998), the European Group for the Study of Insulin Resistance (EGIR) (Balkau and Charles, 1999), the National Cholesterol Education Program’s Adult Treatment Panel III (NCEP: ATP III) (Expert Panel on Detection et al., 2001) and the International Diabetes Federation (IDF) (Alberti et al., 2006), the essential characteristics include obesity, glucose intolerance, dyslipidemia and hypertension (Eckel et al., 2005; McCracken et al., 2018) (Table 1). These characteristics of MetS may contribute to insulin resistance, oxidative stress, inflammation and endothelial dysfunction, which are pivotal mechanisms associated with the pathogenesis of atherosclerosis. Therefore, the regulation of MetS is essential for preventing the progression of atherosclerosis.

**TABLE 1:** Definitions of metabolic syndrome.

Characteristics	WHO 1999	EGIR 1999	NCEP: ATP III 2001	IDF 2006
Basic elements	Glucose intolerance, IGT or diabetes mellitus and/or insulin resistance **plus 2 or more of the following**:	Plasma insulin concentration >75th percentile of non-diabetic patients **plus 2 or more of the following:**	**3 or more of the following:**	Central obesity **plus any 2 of the following:**
Obesity	Waist-to-hip ratio of 0.90 (men) or 0.85 (women) and/or BMI > 30 kg/m^2^	Waist circumference > 94 cm (men) or 80 cm (women)	Waist circumference > 102 cm (men) or 88 cm (women)	Waist circumference* (ethnicity specific) or BMI > 30 kg/m^2^
Fasting plasma glucose	Impaired fasting glucose	≥6.1 mmol/l (110 mg/dl) but non-diabetic	≥5.6 mmol/l (100 mg/dl)	≥5.6 mmol/l (100 mg/dl) or previously diagnosed T2DM
Dyslipidemia	TG ≥ 1.7 mmol/l (150 mg/dl); HDL-C < 0.9 mmol/l (35 mg/dl) (men) or <1.0 mmol/l (39 mg/dl) (women)	TG ≥ 1.7 mmol/l (150 mg/dl) or on treatment; HDL-C < 1.0 mmol/l (39 mg/dl) (men and women)	TG ≥ 1.7 mmol/l (150 mg/dl) HDL-C < 1.7 mmol/l (40 mg/dl) (men); <1.29 mmol/l (50 mg/dl) (women)	TG ≥ 1.7 mmol/l (150 mg/dl) or on treatment HDL-C < 1.03 mmol/l (40 mg/dl) (men) of <1.29 mmol/l (50 mg/dl) (women) or on treatment
Hypertension	≥140/90 mmHg	≥140/90 mmHg	Systolic ≥ 130 mmHg or diastolic ≥ 85 mmHg	Systolic ≥ 130 mmHg or diastolic ≥ 85 mmHg or on treatment
Others	Urinary albumin excretion rate ≥ 20 μg/min or albumin/creatinine ≥ 20 mg/g			

*Waist circumference: for European populations, >94 cm (men) and >80 cm (women); for South Asian, Chinese and Japanese populations, >90 cm (men) and >80 cm (women); for ethnic South and Central American populations, use the South Asian data; and for sub-Saharan African, Eastern Mediterranean and Middle Eastern (Arab) populations, use the European data.

Autophagy is a lysosomal degradation process that plays an important role in maintaining cellular metabolic homeostasis. Previous studies have demonstrated that impaired autophagy is associated with metabolic disorders such as T2DM and MetS via inflammatory pathways and various metabolic stresses (Xu et al., 2016; Kitada et al., 2017; Turkmen, 2017). Autophagy exerts both protective and detrimental effects on cardiovascular disorders. In the progression of atherosclerotic plaques, basal and adaptive autophagy may reduce oxidative stress, inflammation and lipid accumulation and delay the formation of plaques. However, excessive autophagy may cause cell death and plaque instability (Kitada et al., 2016; Luo et al., 2016; Zhu et al., 2017). Therefore, maintaining autophagic homeostasis in cells may be a therapeutic strategy for the treatment of atherosclerosis.

In this review, we discuss the role of autophagy and MetS characteristics in the pathogenesis of atherosclerosis and potential therapeutic drugs that target these molecular mechanisms.

## Metabolic Syndrome Characteristics and the Formation of Atherosclerotic Plaques

The prevalence of MetS is increasing worldwide. In the nearly an decade from 2003 to 2012, the overall prevalence of MetS increased by 1.2% (from 32.9 to 34.7%) in the United States based on the NCEP: ATP III criterion (Aguilar et al., 2015). According to a systematic review summarizing 18 studies, despite differences in methodology, diagnostic criteria and the ages of subjects, nearly 1/5th of the adult population or more are affected by MetS, with a particular increase in prevalence in the Asia-Pacific region (Ranasinghe et al., 2017). A cross-sectional study involving 109,551 Chinese adults showed that MetS was closely related to CVD, especially when MetS was defined by the NCEP: ATP III criteria (Li et al., 2019). The characteristics of MetS, including obesity, glucose intolerance, dyslipidemia and hypertension, may contribute to insulin resistance/hyperinsulinemia, the activation of oxidative stress, the accumulation of proinflammatory cytokines, endothelial dysfunction and other pathological mechanisms. These changes may lead to the pathogenesis of atherosclerosis (Eckel et al., 2005).

### Obesity

**FIGURE 1:**
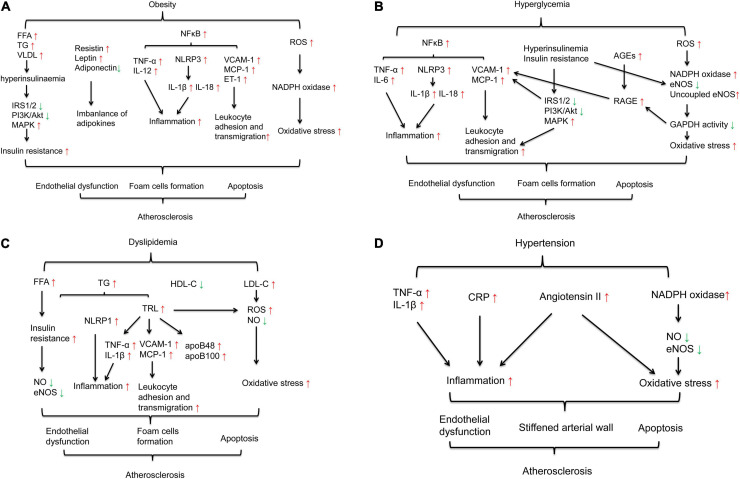
Mechanisms of MetS-induced atherosclerosis. **(A)** Mechanisms of obesity-induced atherosclerosis involvement in insulin resistance, imbalanced adipokines, oxidative stress, and inflammation. **(B)** Mechanisms of hyperglycemia-induced atherosclerosis involvement in inflammation, insulin resistance, the activation of AGEs and oxidative stress. **(C)** Mechanisms of dyslipidemia-induced atherosclerosis involvement in insulin resistance, inflammation and oxidative stress. **(D)** Mechanisms of hypertension-induced atherosclerosis involvement in inflammation, the renin angiotensin system and oxidative stress.

Obesity is a chronic inflammatory disorder characterized by the accumulation of both visceral and subcutaneous fat. Mechanisms of obesity-induced atherosclerosis may involve insulin resistance, an imbalance of adipokines, oxidative stress, inflammation and endothelial dysfunction (Nigro et al., 2006; Lovren et al., 2015) (Figure 1A).

Insulin signaling plays a pivotal role in activating nitric oxide (NO), a vasodilator and antiatherogenic agent, to maintain endothelial function (Zeng and Quon, 1996; Zeng et al., 2000). Typically, insulin binds to the insulin receptor, resulting in tyrosine phosphorylation of insulin receptor substrate-1/2 (IRS-1/IRS-2) and the activation of phosphatidylinositol 3-kinase (PI3K) and protein kinase B (Akt), subsequently augmenting glucose transport and other metabolic processes (Di Pino and DeFronzo, 2019). The administration of endothelin-1 (ET-1), a vasoconstrictor, leads to insulin resistance, as characterized by a decrease in IRS-1 protein levels and suppressed PI3K/Akt activation in rat skeletal muscle (Wilkes et al., 2003) and adipocytes (Ishibashi et al., 2001), further promoting increased vasoconstriction and atherogenesis. In obese conditions, adipose tissue, liver, and skeletal muscle are considered key organs associated with insulin resistance (McArdle et al., 2013). Circulating free fatty acids (FFAs) are released from adipose tissue. In the liver, FFAs increase the production of hepatic glucose and triglycerides (TGs) and induce the secretion of very low-density lipoproteins (VLDLs), which are atherogenic. In skeletal muscle, FFAs reduce insulin sensitivity by inhibiting PI3K activation. Increasing FFAs induces pancreatic insulin secretion, resulting in compensatory hyperinsulinemia and exacerbating insulin resistance (Eckel et al., 2005; McCracken et al., 2018). Moreover, FFA-induced hyperinsulinemia stimulates the mitogen-activated protein kinase (MAPK) pathway and increases reactive oxygen species (ROS) levels and proinflammatory and prothrombotic mediator production via nicotinamide adenine dinucleotide phosphate (NADPH) oxidase stimulation, linking insulin resistance, oxidative stress and inflammation (Satish et al., 2019).

Adipose tissue is the main source of anti-inflammatory and proinflammatory adipokines. Imbalances in these adipokines may contribute to insulin resistance and endothelial dysfunction, leading to atherosclerosis (Lovren et al., 2015). Previous research has shown that resistin, a proinflammatory adipokine, can induce the expression of inflammatory cytokines such as tumor necrosis factor-α (TNF-α) and interleukin-12 (IL-12) in macrophages in a nuclear factor-κB (NF-κB)-dependent manner to promote foam cell formation (Silswal et al., 2005). Moreover, resistin increases the expression of vascular cell adhesion molecule-1 (VCAM-1), monocyte chemoattractant protein (MCP-1) and ET-1 in ECs (Verma et al., 2003). These link resistin to obesity-induced atherosclerosis. Another important adipokine is leptin. Obese individuals exhibit enhanced circulating leptin levels but fail to increase energy expenditure and reduce food intake due to leptin resistance. Two mouse models are widely used to study diabetes- and obesity-associated atherosclerosis: ob/ob mice, which have a mutation in the leptin-encoding gene, and db/db mice, which encode the leptin receptor (Wu and Huan, 2007). Leptin can also stimulate the production of proinflammatory cytokines (Francisco et al., 2018). Adiponectin is an anti-inflammatory adipokine that can directly upregulate insulin sensitivity (Kadowaki et al., 2006; Yamauchi and Kadowaki, 2013). Adiponectin can directly stimulate the production of NO via PI3K-dependent pathways in ECs to mediate vasodilator actions (Chen et al., 2003). In ApoE^–/–^ mice, adiponectin attenuated serum TC, TG and LDL-C levels induced by a high-fat diet, reduced the gene expression of TNF-α, interleukin-6 (IL-6), and VCAM-1, suppressed the activation of the NF-κB pathway, and ultimately inhibited the formation of atherosclerotic plaques (Wang et al., 2016). Another study showed that the association of adiponectin with T-cadherin can protect against neointima proliferation and atherosclerosis (Fujishima et al., 2017).

In both obese human and mouse models, elevated levels of FFAs and fat accumulation increase systemic oxidative stress (Furukawa et al., 2004; Hansel et al., 2004). Oxidative stress results from an imbalance between the production of ROS and antioxidant defenses (Le Lay et al., 2014). In obese mice and adipocytes, increased ROS production is due to an increase in NADPH oxidase. Treatment with NADPH oxidase inhibitors reduces ROS production (Hansel et al., 2004). Elevated ROS can induce nuclear translocation of the NF-κB p65 subunit, activating downstream inflammatory genes and increasing the expression of intercellular adhesion molecule-1 (ICAM-1) and VCAM-1 in ECs (Jayakumar et al., 2014; Medda et al., 2015). Another important oxidative biomolecule is oxidized low-density lipoprotein (oxLDL), which is also elevated in obese individuals (Srikanthan et al., 2016). Oxidation induced by oxLDL can activate IκB kinase (IKK)/NF-κB and c-Jun N-terminal kinase (JNK), leading to endothelial cell death and dysfunction, which contribute to the development of atherosclerosis (Valente et al., 2014).

Obesity is considered a chronic low-grade inflammation (Saltiel and Olefsky, 2017). Multiple inflammatory cytokines, such as TNF-α, IL-6 and C-reactive protein (CRP), are overproduced in adipose tissue, ECs and macrophages in obese humans and in mouse models (Wellen and Hotamisligil, 2005; Coelho et al., 2013). Chronic activation of the NF-κB pathway in ECs upregulates the levels of inflammation-related genes, such as ICAM-1, VCAM-1, and MCP-1, and proinflammatory cytokines, such as TNF-α, IL-6 and IL-1β, further leading to endothelial dysfunction (Kitada et al., 2016). In addition, inflammasome-mediated processes are important in the development of atherosclerosis (De Nardo and Latz, 2011; Lu and Kakkar, 2014). During obesity, the circulating FFAs palmitate and ceramide lead to the activation of the nucleotide-binding oligomerization domain-like receptor family pyrin domain containing 3 (NLRP3) inflammasome (De Nardo and Latz, 2011). Then, NLRP3 activates the production of the mature forms of IL-1β and IL-18, which participate in insulin resistance (Stienstra et al., 2010; Vandanmagsar et al., 2011; Wen et al., 2011) and accelerate atherosclerotic progression (Satish and Agrawal, 2020).

### Glucose Intolerance

Clinical studies have demonstrated that hyperglycemia is a major predictor of atherosclerosis in both diabetic and non-diabetic subjects (Zhang et al., 2006; Nagareddy et al., 2013; Gan et al., 2019). High glucose (HG) may damage arterial cells and play an important role in the progression of atherogenesis. The mechanisms of HG-induced atherosclerosis may involve interactions among insulin resistance, inflammation, advanced glycation end products (AGEs) and oxidative stress, ultimately leading to endothelial dysfunction (Figure 1B).

Insulin resistance is a characteristic feature of T2DM and is usually accompanied by compensatory hyperinsulinemia (Roden and Shulman, 2019). Under diabetic conditions, insulin signaling is impaired at the level of IRS-1, leading to decreased glucose transport and metabolism, impaired endothelial nitric oxide synthase (eNOS) activation and endothelial dysfunction. With increasing concentrations of glucose, the PI3K/Akt pathway is suppressed, leading to proliferative dysfunction in ECs (Varma et al., 2005). Moreover, the MAPK pathway is activated by compensatory hyperinsulinemia, subsequently inducing the expression of VCAM-1 and monocyte adhesion. Insulin resistance suppresses the PI3K/Akt pathway and induces the MAPK pathway to promote endothelial dysfunction and proatherosclerotic events in ECs (Madonna et al., 2004; Di Pino and DeFronzo, 2019).

Another mechanism of HG-induced plaque formation involves activation of the inflammatory/inflammasome pathway. The endothelium is sensitive to changes in glucose concentrations. HG promotes leukocyte adhesion to endothelial cells, which is an initial step in atherogenesis. In human aortic endothelial cells, short-term HG incubation (no more than 12 h) may increase the levels of some adhesion molecules, such as VCAM-1 and MCP-1, via protein kinase C (PKC) and/or NF-κB pathway activation (Piga et al., 2007; Azcutia et al., 2010). These adhesion molecules facilitate monocyte adhesion to ECs, and monocytes differentiate into intimal macrophages and accelerate fatty streak formation. Moreover, monocytes incubated in HG exhibit increased expression of cytokines such as IL-1β and IL-6 (Dasu et al., 2007). NLRP3 inflammasome activation is elevated in type 2 diabetic patients (Lee et al., 2013; Chen et al., 2019) and diabetic rodent models (Luo et al., 2014; Hou et al., 2020). Excessive activation of NLRP3 is associated with cardiac inflammation (Luo et al., 2014). The NLRP3 inflammasome also promotes the secretion of mature IL-1β and IL-18 to initiate the recruitment of inflammatory cytokines, leading to atherothrombosis (Satish and Agrawal, 2020). NF-κB can promote the transcription of NLRP3, pro-IL-1β, and pro-IL-18 in the vascular endothelial cells of diabetic rats. Inhibition of NF-κB reduces activation of the NLRP3 inflammasome and mature IL-1β in HG-treated H9c2 cells, which are heart myoblasts, ameliorating cardiac inflammation, apoptosis and fibrosis (Luo et al., 2014).

HG-induced AGEs are also key proatherogenic mediators in diabetes (Bornfeldt and Tabas, 2011). AGE-modified proteins and lipoproteins can bind to and activate their receptors, such as receptor for AGEs (RAGE). RAGE is expressed in ECs and promotes VCAM-1 expression (Harja et al., 2009). Deletion of RAGE attenuates leukocyte recruitment and protects against atherosclerosis by reducing oxidative stress and decreasing the expression of proinflammatory markers, including NF-κB p65, VCAM-1, and MCP-1, in diabetic ApoE^(–/–)^ mice (Soro-Paavonen et al., 2008).

HG-mediated oxidative stress has been shown to accelerate the progression of atherosclerosis (Giacco and Brownlee, 2010; Katakami, 2018). In the context of diabetes, mitochondria exhibit increased ROS production due to impaired electron transport and ROS scavenging (Xu J. et al., 2020). In mitochondria, ROS activate NADPH oxidases, uncouple eNOS, amplify the production of ROS and reduce GAPDH activity. Inhibition of GAPDH activity increases the expression of RAGE and activates the PKC pathway, which links oxidative stress, RAGE and inflammation and contributes to atherosclerosis (Schaffer et al., 2012; Shah and Brownlee, 2016).

### Dyslipidemia

Dyslipidemia in MetS is closely related to obesity and is characterized by hypertriglyceridemia, low levels of high-density lipoprotein cholesterol (HDL-C) and high levels of low-density lipoprotein cholesterol (LDL-C) (Eckel et al., 2005). Accumulating evidence has demonstrated that hypertriglyceridemia is strongly associated with increased risk of atherosclerosis (Do et al., 2013; Jørgensen et al., 2013; Thomsen et al., 2014). The accumulation of toxic lipid metabolites in muscle, liver, adipocytes and arterial tissues contributes to insulin resistance and endothelial dysfunction and accelerates atherosclerosis. With increases in circulating FFAs released from adipose tissue and transported into the liver, hepatic TG synthesis increases. Hypertriglyceridemia is also a reflection of insulin resistance (Eckel et al., 2005). TG-rich lipoproteins (TRLs) of hepatic origin, such as apolipoprotein B (apoB) 48 and apoB 100, are related to atherosclerosis and are found in plaques (Pal et al., 2003). Dyslipidemia-induced atherosclerosis may be related to multiple mechanisms, including insulin resistance (as mentioned in the obesity section), elevated ROS, and inflammation, leading to endothelial dysfunction (Peng et al., 2017).

Endogenous NO is a signaling molecule that has antiatherosclerotic effects. NO inhibition by excess ROS is the main cause of endothelial dysfunction (Higashi et al., 2009; Bruno et al., 2018). FFAs and TRLs can stimulate intracellular ROS production and cause cellular injury and death in human aortic endothelial cells (Wang et al., 2009). TG accumulation is also related to macrophage oxidative stress, which elevates mitochondrial ROS generation, further promoting foam cell formation (Rosenblat et al., 2012).

Multiple studies have suggested that oxidized FFAs stimulate inflammatory cytokines (Wang et al., 2009; Gower et al., 2011). TRLs upregulate the endothelial expression of ICAM-1 and VCAM-1, facilitate the monocyte infiltration and enhance the endothelial inflammation (Wang et al., 2013). TRL remnants can induce endothelial cell apoptosis and vascular injury by increasing the secretion of cytokines such as IL-1β and TNF-α (Shin et al., 2004). Elevated TG and VLDL were related to arterial inflammation through the NLRP1 inflammasome activation in ECs (Bleda et al., 2016). Low HDL-C is closely related to oxidative stress and insulin resistance (Hansel et al., 2004), which results in endothelial dysfunction through lipotoxicity (Satish et al., 2019) (Figure 1C).

### Hypertension

Blood pressure (BP) levels are strongly correlated with visceral obesity and insulin resistance (Aboonabi et al., 2019). Under insulin resistance/hyperinsulinemia, ET-1 can suppress insulin-induced Akt activation in VSMCs to exacerbate the development of hypertension and atherosclerosis (Lin et al., 2015). Increased systolic BP levels may stiffen the arterial wall and accelerate the progression of atherosclerosis (Mulè et al., 2014; Aboonabi et al., 2019). Hypertension is associated with oxidative stress, increased NADPH oxidase activity, the inactivation of NO, and the downregulation of NO synthase (NOS) isoforms, leading to endothelial dysfunction (Furukawa et al., 2004). Inflammatory cytokines are also pivotal mediators. Increased serum levels of CRP (Sesso et al., 2003), monocyte TNF-α secretion, and serum IL-6 concentrations were reported in patients with hypertension, suggesting a close association between inflammation and hypertension. Moreover, the renin angiotensin system (RAS) plays a major physiological role in endothelial dysfunction and vascular inflammation (Montezano et al., 2014). Studies have demonstrated that angiotensin (Ang) II accelerates the development of atherosclerosis in apoE^–/–^ mice (Daugherty et al., 2000; Weiss et al., 2001). An *in vitro* study also showed that Ang II can induce oxidative stress, inflammation and mitochondrial damage in human umbilical vein endothelial cells (HUVECs), leading to apoptosis and endothelial cell senescence (Dang et al., 2018) (Figure 1D).

## Autophagy in Atherosclerosis

Autophagy is a cellular pathway involved in protein and organelle degradation to maintain cellular metabolic homeostasis (Mizushima et al., 2008). Autophagic dysfunction is closely associated with cancer, neurodegeneration and aging-related diseases such as obesity, diabetes and cardiovascular disorders (Mizushima et al., 2008; Rubinsztein et al., 2011; Kobayashi and Liang, 2015; Kitada et al., 2017). The role of autophagy in atherosclerosis is controversial. On one hand, multiple studies have demonstrated a protective effect of maintaining basal autophagy in atherosclerosis (Kim and Lee, 2014; Nussenzweig et al., 2015; Kim et al., 2018). Characteristics of MetS contribute to impaired autophagy, leading to accumulation of cytotoxic aggregates, dysfunctional organelles (Zhang et al., 2018) and present within the atherosclerotic plaque (Lavandero et al., 2015). Drugs targeting mammalian target of rapamycin (mTOR) signaling showed an effect of stabilizing plaques via repairing impaired autophagy (Ma et al., 2016). On the other hand, although autophagy is critical for maintaining cellular homeostasis under various stress conditions, excessive autophagy may induce autophagy-dependent cell death (Liu and Levine, 2015). MetS-induced reactive ROS, oxidized lipids and inflammation seem to be related to impaired or excessive autophagy activation, contributing to damage to the vascular wall and the development of atherosclerosis.

### Regulatory Mechanisms of Autophagy

**FIGURE 2:**
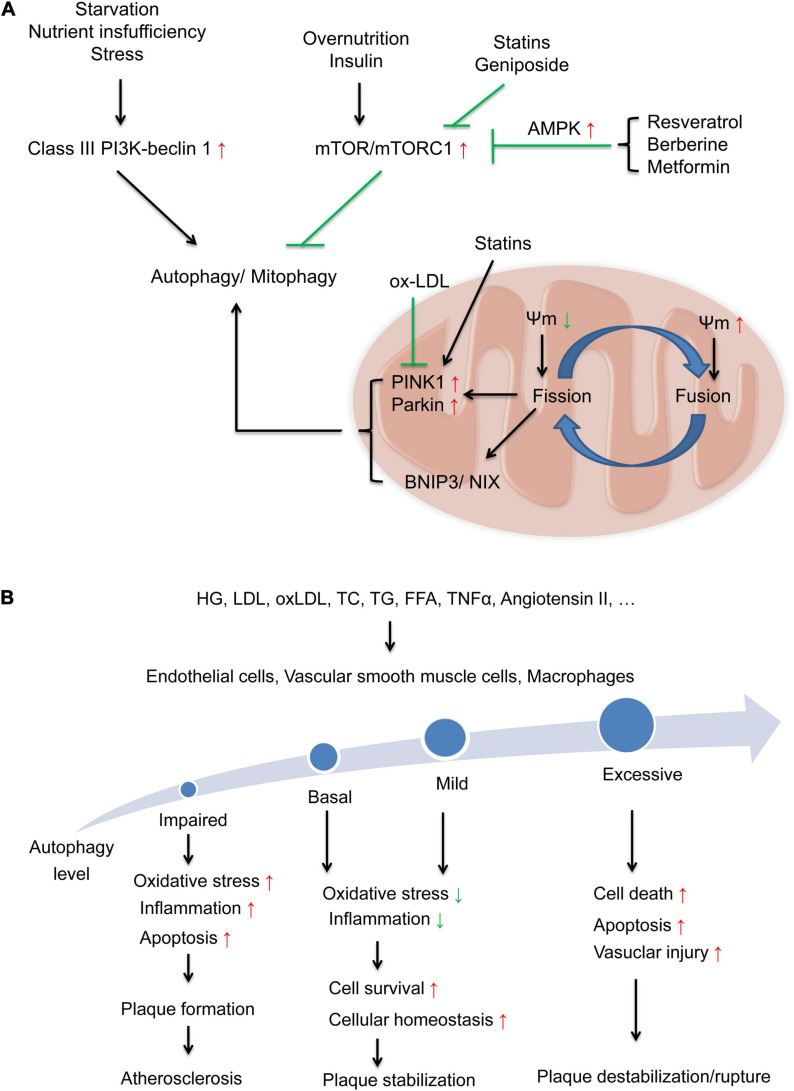
Regulation of autophagy and autophagy levels in different types of cells involved in the progression of atherosclerosis. **(A)** Regulation of autophagy in different states via two major signaling pathways: the inductive pathway mediated by Class-III PI3K-beclin1 signaling and the inhibitory pathway mediated by Class I PI3K-mTOR signaling. Some compounds, such as resveratrol, berberine, metformin, statins and geniposide, may activate autophagy by suppressing the mTOR signaling pathway. Damaged mitochondria are eliminated by mitophagy through the accumulation of PINK1/Parkin pathway and BNIP3/NIX pathway on the mitochondrial surface. With changes in mitochondrial membrane potential (Ψm), this process is coordinated with mitochondrial fusion and fission process. Ox-LDL inhibited PINK1/Parkin then impaired mitophagy and stains activate Parkin-dependent mitophagy. **(B)** Basal and mild adaptive autophagy suppresses oxidative stress and inflammation and increases cell survival and cellular homeostasis to protect against the progression of atherosclerotic plaques, while impaired and excessive autophagy activation leads to increased oxidative stress and inflammation, cell death, and apoptosis, further contributing to plaque instability and rupture.

Autophagy occurs at a basal level and is highly inducible by starvation and other stresses to increase the number of autophagosomes. Autophagosomes enclose misfolded proteins or damaged organelles and then fuse with lysosomes to form autophagolysosomes (Mizushima and Komatsu, 2011). During these processes, multiple autophagy-related genes (Atgs) and proteins are involved (Gatica et al., 2015). Atg1 and microtubule-associated protein 1A/1B-light chain 3 (LC3) are widely regarded as critical markers of autophagy initiation. The conversion of LC3-I to LC3-II causes the formation of autophagolysosomes, and nucleoporin p62 (p62) facilitates the docking of cargo to the cell membrane (Nussenzweig et al., 2015; Hassanpour et al., 2019). The regulatory mechanism of autophagy is closely related to nutritional status. Under conditions of overnutrition or the effects of insulin, class I PI3K is induced to activate mTOR and mTOR complex 1 (mTORC1), thus inhibiting the activation of Atg1. In conditions of nutrient insufficiency, the Class III PI3K-beclin1 complex is triggered to promote the assembly of the Atg12-Atg5-Atg16L complex and Atg8/LC3 and then stimulate autophagosome formation (Shao et al., 2016). In the pathogenesis of atherosclerosis, cavelin-1, a marker protein for caveolar organelles, is involved in the regulation of autophagy. After the formation of phagophore through both mTOR and PI3K pathways, the complex of phagophore and Atg5-Atg12-Atg16 combine with caveolin-1, then interact with LC3 to promote autophagosome formation and facilitate caveolin-1 degradation (Hou et al., 2021). Caveloin-1 deficiency showed elevated Atg7, beclin1 and LC3-II, which indicated an increasing of autophagic activity and atheroprotection (Wu Z. et al., 2019). Characteristics of MetS including glucose (Bai et al., 2020) and dyslipidemia (Chen et al., 2018) inhibited the formation of autophagosomes via activating caveolin-1. Therefore, autophagy may have a close association with the characteristics of MetS and play a key role in the pathogenesis of atherosclerosis (Figure 2A).

### Autophagy in ECs

Endothelial cells are an effective, permeable barrier between circulating blood and tissues (Zhu et al., 2017). MetS-induced autophagy dysregulation has been identified as a critical factor in endothelial dysfunction and atherosclerosis. LDL can suppress endothelial autophagy by activating the PI3K/Akt/mTOR pathway in ECs (Zhu L. et al., 2019). In addition, ox-LDL can inhibit autophagic flux by suppressing the Sirtuin 1 (SIRT 1)/forkhead box protein O1 (FoxO1) pathway to promote apoptosis and adhesion molecule expression in ECs (Wang et al., 2019; Wu Q. et al., 2019). A previous study showed that basal autophagy in ECs induced endothelial eNOS expression and NO bioavailability to maintain endothelial function (Fetterman et al., 2016). Autophagy decreases oxidative stress and inhibits the expression of inflammatory cytokines, including MCP-1 and IL-8. Moreover, inefficient autophagy promotes inflammation and apoptosis and contributes to the development of atherosclerotic plaques in ECs. Impaired autophagy (via Atg3 siRNA) suppresses eNOS phosphorylation and NO production and induces ROS accumulation and inflammatory cytokine production (Bharath et al., 2014). Our previous study also showed that autophagy defects in ECs induced IL-6-dependent endothelial-to-mesenchymal transition and organ fibrosis (Takagaki et al., 2020). High glucose-induced caveolin-1 enhanced LDL transcytosis via autophagic degradation pathway (Bai et al., 2020) and attenuated autophagic flux in response to proatherogenic cytokines (Zhang X. et al., 2020), while caveolin-1 silencing induced autophagy in Human ECs (Bai et al., 2020). This evidence indicates that basal autophagy is a key regulator of oxidant-antioxidant balance and inflammatory-anti-inflammatory balance in ECs.

However, excessive autophagy may mediate cell death in ECs and lead to plaque instability (Martinet and De Meyer, 2009). A previous study indicated that elevated ROS generation caused by oxLDL could induce excessive autophagy characterized by increases in LC3, beclin-1 and Atg5 and apoptosis in ECs, which is a proatherosclerotic characteristic (Ding et al., 2012). Additionally, other research showed that elevated ROS (Shen et al., 2013) and oxLDL (Peng et al., 2014) initiated autophagy in human ECs in an atherosclerotic environment (Figure 2B).

### Autophagy in VSMCs

Abnormalities, death and proliferation in VSMCs participate in the formation and instability of atherosclerotic plaques (Zhang Y. Y. et al., 2020), even lead to vascular neointimal hyperplasia, a central pathogenetic event of post-percutaneous coronary intervention (PCI) restenosis (Zhu and Zhang, 2018). Autophagy is crucial for VSMC function, survival and the development of neointimal hyperplasia in post-PCI restenosis (Zhu and Zhang, 2018). The deficiency of autophagy in VSMCs accelerates cell senescence and promotes diet-induced atherogenesis (Grootaert et al., 2015). The characteristics of MetS have complicated effects on autophagic activity in VSMCs. Previous studies demonstrated that atherosclerotic lesions were markedly increased in high-fat diet-fed ApoE^–/–^ mice and mice with VSMC-specific Atg7 deletion compared with ApoE^–/–^ control mice (Masuyama et al., 2018; Osonoi et al., 2018; Nahapetyan et al., 2019). Modest concentrations of oxLDL (10–40 μg/ml) (Ding et al., 2013) and excess free cholesterol (Xu et al., 2010) enhanced autophagy in VSMCs, as characterized by elevated levels of beclin-1, LC3-II, and Atg5. Induced autophagy is considered a cellular survival mechanism to prevent the death of VSMCs. All these evidence indicate that basal and modest autophagic activity in VSMCs is a protective mechanism against cell death and maintains plaque stability.

In contrast, excessively activated autophagy may result in VSMC death and plaque destabilization. Severe oxidative stress or inflammation stimulates excessive autophagy. TNF-α induces the expression of LC3-II and beclin1 via the JNK/Akt pathway, leading to VSMC death (Jia et al., 2006). In addition, Ang II increases the production of ROS, increases the levels of LC3-II and beclin-1 and decreases Sequestosome 1 (SQSTM1)/p62 to promote autophagosome formation in rat vascular SMCs, which may also be detrimental (Yu et al., 2014) (Figure 2B).

### Autophagy in Macrophages

Macrophages play pivotal roles in all stages of atherosclerosis. During the formation of atherosclerotic plaques, monocytes in the bone marrow are stimulated by MetS conditions, such as elevated TC and LDL, to enter the blood circulation. Circulating monocytes move into the subendothelium of vessel walls and differentiate into macrophages, subsequently turning into foam cells that are filled with oxLDL (Tabas and Bornfeldt, 2016).

The suppression of autophagy in macrophages may lead to apoptosis and plaque destabilization. Macrophage Atg5 deficiency increases apoptosis and oxidative stress in fat-fed LDL receptor-knockout mice and promotes plaque necrosis (Liao et al., 2012). Macrophage-specific Atg5-knockout mice exhibit increased p62 levels and decreased LC3 levels, which are characteristic of autophagy deficiency. Moreover, Atg5-null macrophages secrete IL-1β, leading to inflammasome activation and increased plaques (Razani et al., 2012). Another study demonstrated that macrophage autophagy could be induced by Akt inhibitors, mTOR inhibitors and mTOR-siRNA, while PI3K inhibitors had the opposite effect, which indicates that activating autophagy of macrophage via the inhibition of the PI3K/Akt/mTOR pathway can stabilize vulnerable atherosclerotic plaques (Zhai et al., 2014). This evidence suggests an atheroprotective role for basal autophagy in macrophages.

However, excessive autophagy may also lead to autophagic death in macrophages via poorly understood type II programmed cell death, which further exacerbates the inflammatory response (Liu and Levine, 2015; Hassanpour et al., 2019). Future studies are necessary to identify the detrimental role of autophagy in macrophages (Figure 2B).

### Mitophagy

Mitochondrial dynamics, including mitochondrial fusion, fission, biogenesis and mitochonial autophagy (mitophagy) can be regulated by the characteristics of MetS (Vásquez-Trincado et al., 2016). Cells selectively remove dysfunctional and damaged organelles via mitophagy. Briefly, the process of mitophagy is mainly regulated by PTEN-induced kinase 1 (PINK1) and Parkin proteins. Damaged mitochondria are eliminated by mitophagy through the accumulation of PINK1 and Parkin on the mitochondrial surface. With changes in mitochondrial membrane potential (Ψm), this process is coordinated with mitochondrial fusion and fission process. Pro-apoptotic BH3-only domain protein (BNIP3) and NIX are also involved in the selective mitochondrial clearance (Ashrafi and Schwarz, 2013; Vásquez-Trincado et al., 2016) (Figure 2A). Deregulation of mitophagy leads to accumulation of dysfunctional and damaged mitochondria, results in the overload of ROS, depletion of adenosine triphosphate (ATP) and apoptosis of cardiomyocytes, which may lead to the pathogenesis of CVD including atherosclerosis (Chistiakov et al., 2018; Morciano et al., 2020).

The molecular mechanism mediating mitophagy in the pathogenesis of atherosclerosis may involve mitochondrial fission, accumulation of PINK1 and the recruitment of Parkin to mitochondria. Multiple studies demonstrated that characteristics of MetS, especially obesity and dyslipidemia impaired mitochondrial dynamic and mitophagy. Ox-LDL decreased mitochondrial aldehyde dehydrogenase 2 (ALDH2) via ROS-mediated VSMCs senescence (Zhu H. et al., 2019), caused endothelial apoptosis via inhibiting fusion and mitophagy (Zheng and Lu, 2020). Ox-LDL inhibited PINK1 and Parkin then impaired mitophagy flux, which leads to VSMC apoptosis (Swiader et al., 2016). Moreover, another research showed that SIRT3/FOXO3a/parkin pathway in macrophages is a potential target for suppressing NLRP3 inflammasome activation to attenuate plaque size and vulnerability (Ma et al., 2018).

## Pharmacological Interventions in the Treatment of Atherosclerosis

Based on the effects of MetS characteristics and autophagy on the pathogenesis of atherosclerosis, we suggest that targeted autophagy therapy may be an effective and promising strategy for atherosclerosis treatment. At present, many drugs for the treatment of MetS have additional benefits on autophagy regulation to protect against atherosclerosis. The underlying mechanisms of these drugs are related to the inhibition of the mTOR signaling pathway, oxidative stress, inflammation or hyperlipidemia (Figure 2A and Table 2).

**TABLE 2:** Antiatherosclerotic compounds and mechanisms.

Compounds	Mechanisms of autophagy induction	Primary functions	Antiatherosclerotic effects
Resveratrol	AMPK activation, mTOR inhibition, anti-inflammation, antioxidation, SIRT1 activation	AMPK activation	Decreases the size and density of atherosclerotic plaques, reduces the layer thickness (Wang et al., 2005)
Metformin	AMPK activation, mTOR inhibition, anti-inflammation, antioxidation, anti-hyperlipidemia	Anti-hyperglycemia	Reduces monocyte-to-macrophage differentiation (Vasamsetti et al., 2015), promotes cholesterol efflux, attenuates plaque formation, and decreases atherosclerotic lesion areas (Luo et al., 2017)
Statins	mTOR inhibition, anti-inflammation	Anti-hyperlipidemia	Plaques stabilization (Bea et al., 2002; Rodriguez et al., 2017), reduces infarct size (Andres et al., 2014)
Berberine	AMPK activation, mTOR inhibition, anti-inflammation, antioxidation, anti-hyperlipidemia	AMPK activation	Inhibition of inflammation in macrophages (Fan et al., 2015)
Geniposide	mTOR inhibition	Anti-inflammation	Decreases the size of atherosclerotic plaques (Xu Y. L. et al., 2020)

### Resveratrol

Resveratrol, an activator of 5’-adenosine monophosphate-activated protein kinase (AMPK), is a polyphenolic phytoalexin that occurs naturally in many plant parts and products. Resveratrol has been verified to have antidiabetic (Kitada et al., 2011; Szkudelski and Szkudelska, 2015) and cardiovascular benefits (Bonnefont-Rousselot, 2016). Resveratrol treatment results in a decrease in the size and density of atherosclerotic plaques and a reduction in layer thickness in a rabbit model (Wang et al., 2005). Resveratrol prevents high-fat/sucrose diet-induced central arterial wall inflammation and stiffening in a monkey model (Mattison et al., 2014). In addition to directly activating autophagy by inhibiting mTOR (Sanches-Silva et al., 2020), the underlying mechanisms include the indirect activation of autophagy via anti-inflammatory and antioxidant effects. Resveratrol is the most well-known compound that stimulates members of the sirtuin family. Our previous research showed that sirtuin 1 (SIRT1) inactivation induces inflammation through NF-κB activation and dysregulates autophagy via mTOR/AMPK pathways in THP-1 cells, a human monocyte cell line (Takeda-Watanabe et al., 2012), which indicated the relationship between autophagy impairment and inflammation. Another *in vitro* study showed that resveratrol enhanced autophagic flux and promoted ox-LDL degradation in HUVECs (Zhang et al., 2016) and macrophages (Liu B. et al., 2014) via the upregulation of SIRT1. Resveratrol also attenuates EC inflammation by inducing autophagy in part via the activation of the AMPK/SIRT1 pathway (Chen et al., 2013) and SIRT1/FoxO1 pathway (Wu Q. et al., 2019). Moreover, resveratrol can promote autophagosome formation characterized by LC3 production and p62 degradation and suppress palmitic acid-induced ROS to attenuate endothelial oxidative injury in HUVECs (Zhou et al., 2019).

### Metformin

Metformin is the recommended first-line treatment for T2DM. Beyond its antidiabetic effects, the benefits of metformin on MetS and cardiovascular diseases have also been confirmed (Zhou et al., 2018). As an inducer of AMPK, the underlying mechanisms of metformin may contribute to stimulating autophagy via the AMPK pathway and exert anti-inflammatory, antihyperlipidemic and antioxidant effects.

Metformin can directly activate AMPK and then suppress the mTOR pathway to induce autophagy and inhibit atherosclerosis (You et al., 2020). An *in vitro* study showed that metformin inhibits IL-1β, IL-6, and IL-8 in ECs, VSMCs, and macrophages by blocking the PI3K-Akt/NF-κB pathway (Isoda et al., 2006). In addition, metformin reduces monocyte-to-macrophage differentiation and attenuates Ang II-induced atherosclerotic plaque formation in ApoE^–/–^ mice by decreasing AMPK activity to suppress the phosphorylation of signal transducer and activator of transcription 3 (STAT3) (Vasamsetti et al., 2015). Given its antihyperlipidemic effects, metformin protects against ox-LDL-induced lipid uptake and apoptosis in macrophage (Huangfu et al., 2018), prevents TC uptake during oxidative stress-induced atherosclerosis (Gopoju et al., 2018). Combination therapy with metformin and atorvastatin decreased the atherosclerotic lesion areas in rabbits fed a high-cholesterol diet. In macrophages, this cotreatment promoted cholesterol efflux to achieve antiatherosclerotic benefits (Luo et al., 2017). In terms of antioxidant effects, metformin reduces NADPH oxidase and increases antioxidative enzymes such as superoxide dismutase (SOD), glutathione peroxidase and catalase in cultured human monocytes/macrophages, which alter the oxidative status of macrophages and increases antioxidative activity (Bułdak et al., 2016).

### Statins

Statins are the cornerstone for the prevention of atherosclerotic cardiovascular disease (Rodriguez et al., 2017). Statins exert stabilizing effects on vulnerable atherosclerotic plaques in both clinical research (Rodriguez et al., 2017) and animal models (Bea et al., 2002). Beyond the hypolipidemic effects, stains are considered autophagy inducers via mTOR inhibition, mediating anti-inflammatory elements to protect against atherosclerosis (Mizuno et al., 2011; Martinet et al., 2014). In macrophages, atorvastatin inhibits LPS-induced inflammatory factors such as IL-1β and TNF-α by enhancing autophagy through the Akt/mTOR signaling pathway (Han et al., 2018). In VSMCs, atorvastatin protects against transforming growth factor-β1 (TGF-β1)-induced calcification by stimulating autophagy (Liu D. et al., 2014). Moreover, atorvastatin can reverse the endothelial cell dysfunction induced by Ang II (Dang et al., 2018). Simvastatin, another kind of statin, inhibits the mTOR pathway to increase autophagy (Wei et al., 2013) and activates Parkin-dependent mitophagy (Andres et al., 2014) in cardiomyocytes. This evidence highlights the role of statins in the treatment of atherosclerosis.

### Natural Products

Similar to resveratrol, berberine, an extract of Coptis, exhibits antioxidant, anti-inflammatory, and antihyperlipidemic effects (Tillhon et al., 2012). A previous study showed that berberine suppressed ox-LDL-induced inflammation and increased the conversion from LC3-I to LC3-II in macrophages through the activation of the AMPK/mTOR pathway (Fan et al., 2015).

Geniposide, an extract of *Gardenia jasminoides Ellis*, shows antioxidant and anti-inflammatory effects (Fu et al., 2012). Previous study demonstrated that geniposide decreased the size of atherosclerotic plaques, inhibited the progression of atherosclerosis in high fat diet-fed ApoE^–/–^ mice. The potential mechanism may contribute to the reinforce of macrophage autophagy by inhibiting the triggering receptor expressed on myeloid cell 2 (TREM2)/mTOR signaling (Xu Y. L. et al., 2020).

## Conclusion

The characteristics of MetS are closely related to oxidative stress, inflammation, insulin resistance, and imbalanced adipokines and are responsible for both impaired and excessive autophagy. Autophagy homeostasis is the key regulator of MetS-induced atherosclerosis. Dysregulation of autophagy induced by MetS contributes to endothelial dysfunction, monocyte/macrophage migration and adhesion that lead to the progression of atherosclerosis. Basal and mild adaptive autophagy protect against the progression of atherosclerotic plaques, while impaired autophagy or excessive autophagy activation induced by MetS is related to oxidative stress, inflammation, apoptosis, and foam cell formation, contributing to plaque instability or even plaque rupture. Presently, multiple drugs used to treat MetS have been indicated to regulate autophagy beyond their fundamental effects. Given the double-edged sword effect of autophagy, precise control of autophagy should be considered a potential therapeutic strategy in the prevention and treatment of atherosclerosis.

## Author Contributions

JX contributed to drafting and writing the article. MK, YO, and DK contributed to the discussion of the review. All authors revised the manuscript critically for important intellectual content and approved the final version to be published. MK is responsible for the integrity of the content.

## Conflict of Interest

The authors declare that the research was conducted in the absence of any commercial or financial relationships that could be construed as a potential conflict of interest.
